# Ionospheric Correction Based on Ingestion of Global Ionospheric Maps into the NeQuick 2 Model

**DOI:** 10.1155/2015/376702

**Published:** 2015-03-01

**Authors:** Xiao Yu, Chengli She, Weimin Zhen, Nava Bruno, Dun Liu, Xinan Yue, Ming Ou, Jisheng Xu

**Affiliations:** ^1^School of Electronic Information, Wuhan University, No. 129 Luoyu Road, Wuhan 430079, China; ^2^China Research Institute of Radiowave Propagation, No. 36 Xianshandong Road, Qingdao 266107, China; ^3^University of Chinese Academy of Sciences, Beijing 100029, China; ^4^Key Laboratory of Earth and Planetary Physics, Institute of Geology and Geophysics, Chinese Academy of Sciences, Beijing 100029, China; ^5^The Abdus Salam International Center for Theoretical Physics, T-ICT4D Laboratory, 34100 Trieste, Italy; ^6^The Constellation Observing System for Meteorology, Ionosphere, and Climate Program Office, University Corporation for Atmospheric Research, Boulder, CO 80303, USA

## Abstract

The global ionospheric maps (GIMs), generated by Jet Propulsion Laboratory (JPL) and Center for Orbit Determination in Europe (CODE) during a period over 13 years, have been adopted as the primary source of data to provide global ionospheric correction for possible single frequency positioning applications. The investigation aims to assess the performance of new NeQuick model, NeQuick 2, in predicting global total electron content (TEC) through ingesting the GIMs data from the previous day(s). The results show good performance of the GIMs-driven-NeQuick model with average 86% of vertical TEC error less than 10 TECU, when the global daily effective ionization indices (Az) versus modified dip latitude (MODIP) are constructed as a second order polynomial. The performance of GIMs-driven-NeQuick model presents variability with solar activity and behaves better during low solar activity years. The accuracy of TEC prediction can be improved further through performing a four-coefficient function expression of Az versus MODIP. As more measurements from earlier days are involved in the Az optimization procedure, the accuracy may decrease. The results also reveal that more efforts are needed to improve the NeQuick 2 model capabilities to represent the ionosphere in the equatorial and high-latitude regions.

## 1. Introduction

The ionosphere, the ionized part of the atmosphere extending from ~60 to several thousand kilometers above the Earth surface, can affect the radiowave signals travelling through it in different ways, such as Faraday rotation, doppler frequency shift, ray path bending, carrier phase advance and pseudorange group delay, and fluctuations of signal intensity and phase (ionospheric scintillation) [1–4]. Regarding the L band of the Global Navigation Satellite System (GNSS) signal, the major influence of the ionosphere is the carrier phase advance and pseudorange group delay on ranging signals depending on the ionospheric total electron content (TEC, Unit: TECU, 1 TECU = 10^16^ el/m^2^). For code measurements, the consequent pseudorange delay due to the ionosphere Ig[m] can be described as a first approximation by

(1) $$\begin{array}{c} I_{g}=\frac{40.3}{f^{2}}\times \text{sTEC}. \end{array}$$

Here, the slant TEC (sTEC) is defined as the integral of the electron density along the path from the transmitter to the receiver. As it is well known, the GNSS single frequency receivers have to compensate for the unwanted term Ig, before solving the navigation equations. In this case an explicit estimate of the TEC is usually obtained by means of an ionospheric model.

Several models that could be used to calibrate the ionospheric term have been developed and are still hot topics of investigation for navigation-related applications. The GPS ionospheric correction algorithm (ICA), known as Klobuchar model [5], designed on the basis of the Bent model, introduces many geometric approximations aiming at reducing the receiver computational load. The Klobuchar model provides a daily vertical TEC (vTEC) profile consisting of a cosine representation during the day and a constant value during the night. Both the amplitude and period of the cosine term are represented by four broadcast coefficients defining a third order polynomial of the geomagnetic latitude. The phase of the maximum is fixed at 14:00 local time. Through a thin shell ionosphere assumption, a suitable mapping function is applied to convert the vertical time delay at the pierce point to a slant delay along a given ray path. The GPS ICA is supposed to provide a 50% correction of the ionospheric time delay and the interested reader is referred to [5] for a detailed description of the algorithm.

The Galileo ICA can be described as follows. (1) The effective ionization parameter (Az) at each monitoring station is determined through minimizing the differences between observed and NeQuick modeled slant TEC values for the given day (it is assumed that Az will be valid for the following day). (2) From the calculated Az at different monitoring stations, a global Az is determined as a function of Modip using a 2nd degree polynomial determining a set of 3 coefficients. Then the satellite transmits the relevant Az in the navigation message in terms of 3 coefficients. (3) The receiver calculates slant TEC using NeQuick with the broadcast ionization parameters and corrects the ionospheric delay at the specific frequency [6–10].

Some excellent work about the performance of the Galileo-like model in providing the ionospheric delay predictions has been published [7–10]. In these assessment studies, some IGS stations were used as reference stations to create the broadcast message and the others were used as test stations to obtain the slant TEC mismodeling. In [7], the performance in the equatorial region and northern mid-latitude region during May 2000 is given by the probability density function of residual error. In [8], a comparison of the results obtained by the GPS and Galileo operational models with observations is presented in terms of the year 2000 daily 65 percentile and 95 percentile of the absolute values of the mismodelings. In [9], the performance of the NeQuick model in correcting the ionospheric delay was obtained by comparing its predictions with Topex/Poseidon TEC data of 3 March 2000. In [10], slant TEC data for the year 2002 were ingested into NeQuick for a dozen locations around the world where colocated ionosonde and GPS receiver allow comparing measured and modeled TEC values. The dataset used by these studies covers a single day, a whole month or year during high solar activity, and their results indicate that the performance of Galileo ICA is better than GPS ICA and can be used to correct the observed ionospheric delay in a realistic way.

In the present work, a long history (over 13 years) of global ionospheric maps (GIMs) is adopted as the primary source of data to investigate the capabilities of NeQuick 2 model in providing global daily TEC prediction in a statistical way.  Section 2 gives a short description of NeQuick 2 model and dataset.  Section 3 describes the ionospheric correction algorithm based on NeQuick 2 adaption to GIMs in a Galileo-like mode and the criteria used to carry out the assessment.  Section 4 presents and discusses the results and also a test study to improve the model performance further. Conclusions are then drawn in Section 5.

## 2. NeQuick 2 Model and Dataset

NeQuick 2 [11] is the latest version of a quick-run ionospheric electron density model particularly tailored for transionospheric propagation applications, developed at the Aeronomy and Radio Propagation Laboratory (ARPL, now T/ICT4D) of The Abdus Salam International Center for Theoretical Physics (ICTP), Trieste, Italy, and at the Institute for Geophysics, Astrophysics and Meteorology (IGAM) of the University of Graz, Austria.

To describe the electron density of the ionosphere, the NeQuick model uses a DGR profile formulation, which is proposed by di Giovanni and Radicella [12] and subsequently modified by Radicella and Zhang [13] and Radicella and Leitinger [14]. The model describes the ionosphere separately for the bottomside and the topside. The bottomside goes from 60 km to the F2-layer peak and consists of a sum of five semi-Epstein layers. The topside is above the F2 peak layer and it is described by means of a semi-Epstein layer with a height-dependent thickness parameter. To compute the thickness parameters and the peak electron density and height for the Epstein layers, NeQuick employs the ionosonde parameters which can be modeled or experimentally derived. The major changes in the representation of the topside and bottomside in NeQuick 2 can be found from [15, 16].

NeQuick 2 outputs the ionospheric electron density and TEC as well for the given location, time of the day, season, and solar activity indices. In order to improve the model performance and prediction capabilities, data ingestion and assimilation techniques have been implemented [6–10, 17–20], which replace the standard solar activity indices with different “effective” parameters that allow adapting a model to a specific data set. These techniques become part of COST296 Action course [21–23].

In this study, GIMs are chosen as measured values for convenience [24–27]. GIMs are computed based on the International GNSS Service (IGS) network where GPS receivers are distributed worldwide. There are at least five analysis centers that generate and deliver long-term GIMs: CODE, EMR, ESA, JPL, and UPC. Different agencies may use different reference frames and techniques to estimate vTEC and differential code biases (DCB). After computation, three validation centers (JPL, ESA, and UPC) combine them into a common IGS GIM. The global accuracy of this combined TEC is about 2–8 TECU depending on the epoch in the solar cycle, season, latitude, and proximity of available GPS receivers [28].

We choose GIMs computed by JPL (Jet Propulsion Laboratory) and CODE (Center for Orbit Determination in Europe) as measured values. GIMs generated at both agencies use data from more than 100 GPS sites of the IGS and other institutions. At JPL, the vertical TEC is modeled in a solar-geomagnetic reference frame using bicubic splines on a spherical grid, and a Kalman filter is used to solve simultaneously instrumental biases and vTEC on the grid (as stochastic parameters). More details about the JPL GIM algorithm and daily process can be found in [29–31]. At CODE, the vertical TEC is modeled in a solar-geomagnetic reference frame using a spherical harmonics expansion, and piecewise linear functions are used for the representation in the time domain. Daily DCB for all GPS satellites and ground stations are estimated simultaneously as constant values for each day. More details about the CODE GIM algorithm and daily process can be found in [32–34].

At both agencies, the GIMs are computed every 2 hours. From its beginning to 3 November 2002, the GIMs represented global TEC distribution at odd-hours and then they switched to even-hours. The vertical TEC values in the grids are given along the geographic latitude and longitude, from 87.5°S to 87.5°N and 180°W to 180°E, with intervals of 2.5° and 5°, respectively. The period of data used in this paper spans from its beginning to 31 December 2011. The CODE GIMs start from 28 March 1998 and the dataset is perfectly complete and spans 5027 days. The JPL GIMs start from 28 August 1998 and are not available on several days and they span 4856 days.

**Figure 1:**
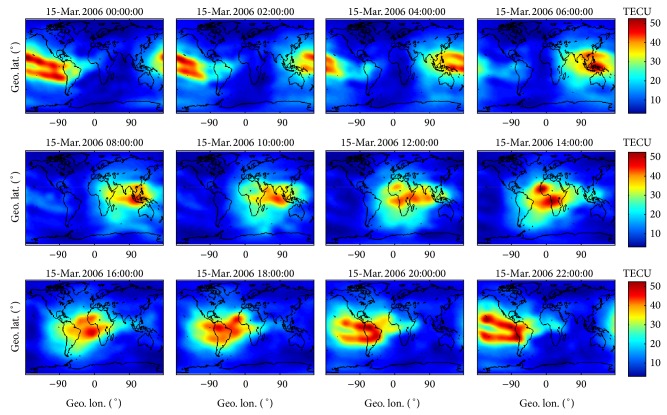
12 JPL GIMs on 15 March 2006. In each panel, the *x*-axis and *y*-axis represent geographic longitude and latitude, respectively, and the color scale indicates the TEC in TECU.

As an example, Figure 1 presents 12 JPL GIMs on 15 March 2006 (*F*10.7 = 73.4, low solar activity), where the features of equatorial anomaly are quite evident. In each panel, the *x*-axis and *y*-axis represent geographic longitude and latitude, respectively, and the color scale indicates the TEC in TECU.

## 3. Ionospheric Correction Algorithm

Considering the criteria expressed in [6–10], an ionospheric correction algorithm based on ingestion of GIMs into NeQuick 2 model has been implemented. To generate the Galileo-like ionospheric correction parameters, the following two procedures are considered. First of all, NeQuick 2 is optimized as a function of the daily effective ionization level (Az) to be adapted to the measured vTEC values. Following the Galileo ICA, Az is applicable for a period of 24 hours forecasting. The root mean square of TEC differences at a given grid point is defined as TEC residual errors which is called ΔTEC. It is calculated as follows:

(2) $$\begin{array}{c} \Delta \text{TEC}=\sqrt{\frac{\left(\sum_{i=1}^{N}{\left({\text{TEC}}_{\text{observed}}-{\text{TEC}}_{\text{modeled}}\left(F10.7\right)\right)}^{2}\right)}{N}}. \end{array}$$

Here, *N* is the number of individual observations during the day and TEC_observed_ and TEC_modeled_ are the TEC values given by GIMs and NeQuick 2, respectively. At a given time and for a given ray path, the TEC value obtained from the integral of NeQuick 2 electron density profile depends monotonously on the solar flux input. Therefore, there should be an optimum solar flux input value, defined as daily effective “ionization level” parameter (Az), to minimize the mismodeling of NeQuick 2 at the grid point.

**Figure 2:**
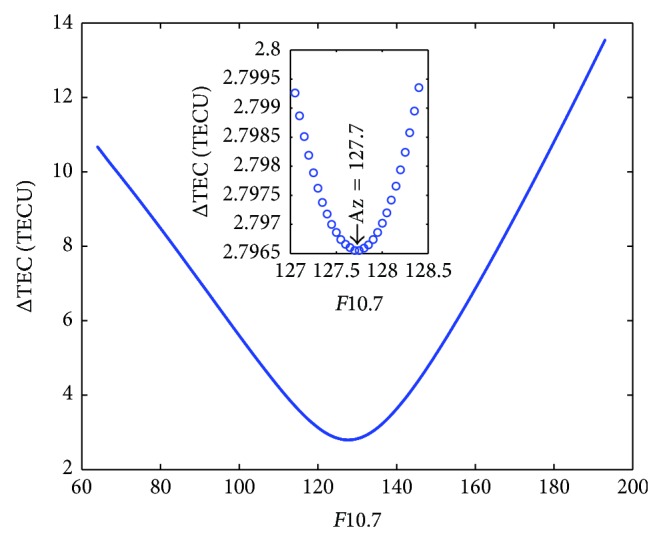
TEC residual error, ΔTEC versus *F*10.7 at a grid point (45°N, 0°E) for 30 May 2004. The TEC observations are from JPL GIMs and the daily Az value for the grid point equals 127.7.

As an example, Figure 2 illustrates how ΔTEC (defined in (2)) changes versus *F*10.7 at a grid point (45°N, 0°E) for a given day (30 May 2004). The TEC observations are from JPL GIMs. It can be seen that ΔTEC decreases from 10.67 TECU to 2.80 TECU when *F*10.7 increases from 63 to 127.7. By contrast, as *F*10.7 increases from 127.7 to 193, ΔTEC increases from 2.80 TECU to 13.54 TECU. Thus the residual error of NeQuick 2, defined as ΔTEC, reaches a minimum when *F*10.7 approaches to 127.7; the daily Az value for the grid point is therefore defined to be 127.7.

**Figure 3:**
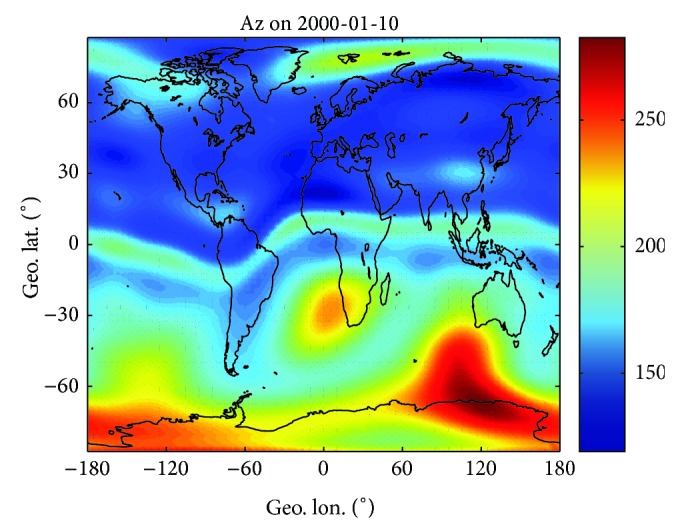
Geographic distribution of Az for 10 January 2000, after ingesting the current day JPL GIMs into the NeQuick 2 model.

**Figure 4:**
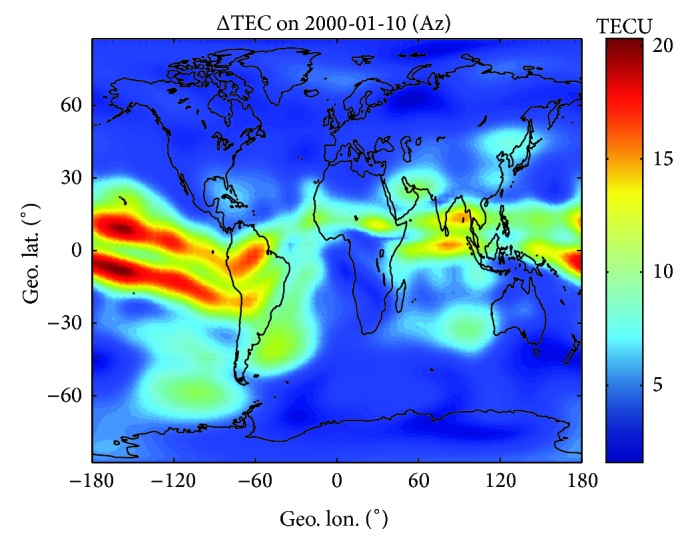
Geographic distribution of TEC residual errors for 10 January 2000, after ingesting the current day JPL GIMs into the NeQuick 2 model.

Repeating the procedures described above, global maps of Az and ΔTEC can be obtained for the selected day. Figures 3 and 4 illustrate the geographic distribution of Az and ΔTEC on 10 January 2000 (*F*10.7 = 157.8, high solar activity), respectively. Following the geographic distribution of both parameters, the world can be divided into 5 wide regions characterized by quite different behaviors.

1. The equatorial region, corresponding to a narrow belt (~15° width) around the geomagnetic equator, which exhibits Az values a little higher than *F*10.7 and largest TEC residual errors. The TEC errors show maxima (close to 20 TECU) in the crests of the equatorial anomaly while being significantly lower at geomagnetic equator.
2. Mid-latitude bands, extending from the equatorial region boundary to about 60° northward and southward of the equator, which show smooth Az values (near to *F*10.7) and low residual errors (no more than 10 TECU). It is interesting to note that both parameters are smoother and lower in the northern hemisphere than in the southern hemisphere, in accordance with the highest number of northern observations incorporated by both NeQuick and GIMs.
3. Two polar regions, extending from about 60° northward and southward of the equator to polar caps, which exhibit the highest (up to 320) and highly variable Az values. However, the TEC residual errors are the lowest (no more than 5 TECU) and more remarkable in the southern hemisphere. The Az peaks around Auroral zone might be partly due to the fact that the enhanced ionospheric ionization in ~120 km altitude resulting from the energetic particles precipitation is not included in the NeQuick empirical modeling [35]. The energetic particles precipitation occurs in spring, fall, and winter. NeQuick 2 and GIMs in polar region may not behave as well as in mid-and-low latitude regions; therefore we only consider dataset having the geographical latitude between 60°S and 60°N in the following study.

**Figure 5:**
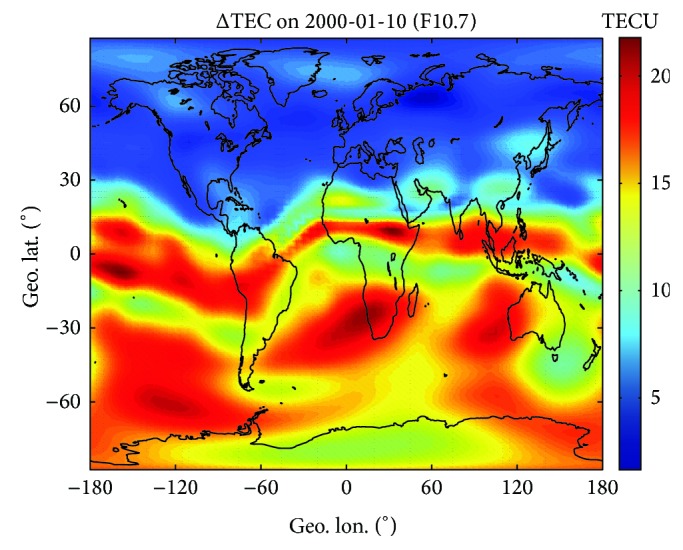
Geographic distribution of TEC residual errors for 10 January 2000, when NeQuick 2 model is driven by *F*10.7.

To demonstrate the effectiveness of data ingestion techniques in adapting NeQuick 2 to GIMs dataset, Figure 5 displays a similar figure as Figure 4, but NeQuick 2 is driven by standard input, the *F*10.7 index of the day. Comparing both figures, there is no remarkable difference of residual error in the northern hemisphere. But it is not the case in the equatorial and southern hemisphere, where much higher residual errors (up to 15–20 TECU) are present. It can be concluded that through designating a much higher (sometimes lower) Az value than real *F*10.7 index, data ingestion process would reduce the discrepancies.

Even if it is not a real ionospheric parameter, Az exhibits variations with geographic location. To describe the mixed dependence of Az on the geomagnetic field and geographical latitude, modified dip latitude (MODIP [36]) *μ* [°] defined as follows has been used:

(3) $$\begin{array}{c} \tan\mu =\frac{I}{\sqrt{\cos\phi }}. \end{array}$$

Here, *I* and *ϕ* denote the geomagnetic dip and geographic latitude, respectively. *I* can be computed using the IGRF (or DGRF) models.

After the daily Az values for all the 3528 grid points (72 and 49 grid points along the geographic longitude and latitude, resp.) are calculated and collected, Az can be regressed as a second order polynomial function of MODIP:

(4) $$\begin{array}{c} \text{Az}=a_{0}+a_{1}\mu +a_{2}\mu^{2}. \end{array}$$

In the following, the three coefficients *a*_0_, *a*_1_, and *a*_2_ will be calculated after an optimization procedure based on the global observations. Then Az_modeled_ will be obtained by substituting *a*_0_, *a*_1_, *a*_2_ and *μ* values into (4). An example illustrated how Az values (denoted as original values, with blue dots) change versus MODIP is shown in Figure 10, together with Az_modeled_ values using (4) (green), and will be discussed later.

For a given grid point, the GIMs-driven-NeQuick results can be obtained by using Az_modeled_ as NeQuick 2 input. Taking the GIMs as reference, a statistical analysis on the global TEC difference (errors) can be carried out to check the ability of NeQuick 2 to reproduce global TEC distribution. Likewise, if 12 GIMs of the previous day are used as ingested dataset and GIMs of the current day are considered as reference, the performance of NeQuick 2 in ionospheric correction prediction (one day in advance) can be obtained through 12 maps of TEC errors for a given day.

**Figure 6:**
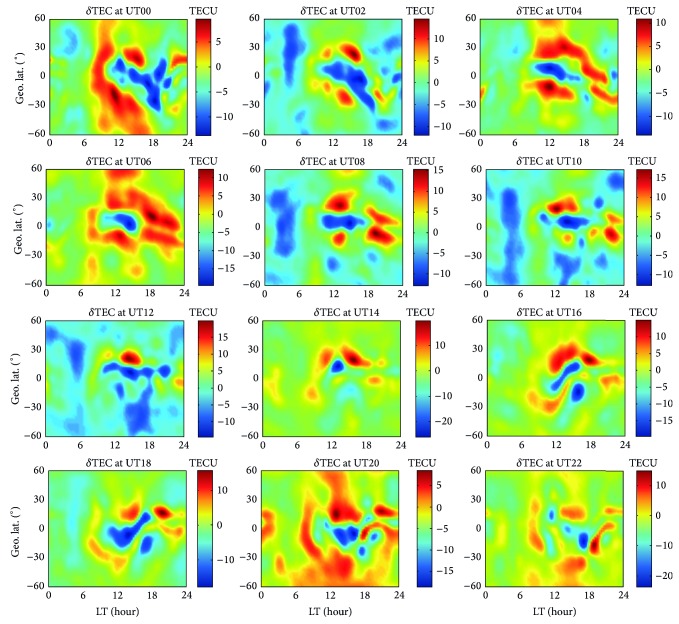
As Figure 1, but for TEC differences, when the previous day JPL GIMs are ingested into NeQuick 2 in a Galileo-like mode. The *x*-axis and *y*-axis denote LT and geographic latitude, respectively.

Figure 6 displays 12 maps of TEC differences on 15 March 2006 after ingesting the previous day JPL GIMs. The *x*-axis and *y*-axis denote LT and geographic latitude, respectively. Indicated from the figure, it is evident that the maximum, minimum, and highest variability of TEC differences (denoted as *δ*TEC) appear during daytime, but their amplitude seldom exceeds 15 TECU. As the time delays on GNSS signals are proportional to the TEC values, which makes the necessity of ionospheric correction not as impendent at nighttime as at daytime, the nighttime situations (LT > 18 or LT < 6) are discarded.

To assess the performance of ionospheric correction based on ingestion of GIMs into NeQuick 2, the cumulative distribution function (CDF) of TEC error absolute values not exceeding a certain value can be taken as a criterion, which is defined as follows:

(5) $$\begin{array}{c} \text{CDF}\left(x\right)=\frac{N_{\text{thr}}\left(x\right)-N_{\text{thr}}\left(-x\right)}{N_{\text{tot}}}. \end{array}$$

**Figure 7:**
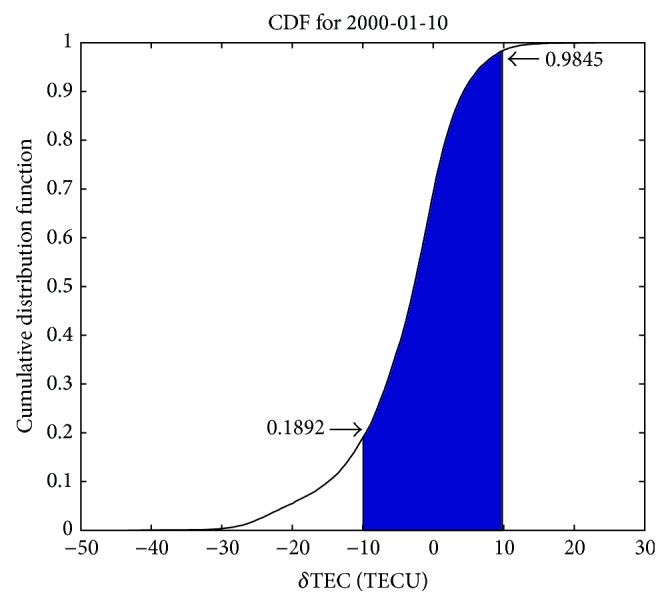
Cumulative distribution function (CDF) of TEC errors for 10 January 2000, when the previous day JPL GIMs are ingested into NeQuick 2 in a Galileo-like mode. The blue area denotes CDF of absolute values of TEC errors less than 10 TECU, and zi_10_ equals 0.7953 (0.9845 − 0.1892).

Here, *N*_tot_ is the number of daytime TEC grid points involved in the data ingesting process, and *N*_thr_(*x*) is the number of grid points where daytime TEC errors are not exceeding a certain value, *x*. Since the absolute values of TEC error seldom exceed 15 TECU, we take 10 TECU as the threshold and denote the parameter (CDF of absolute values of TEC error not exceeding 10 TECU) as zi_10_. An example of cumulative distribution function of TEC errors is given in Figure 7 (for 10 January 2000), when JPL GIMs of the previous day are ingested into NeQuick 2. The blue area denotes CDF of the absolute values of TEC errors less than 10 TECU. Here, computed CDF for TEC errors less than 10 TECU, *N*_thr_(10), and less than −10 TECU, *N*_thr_(−10), are 0.9845 and 0.1892, respectively; therefore zi_10_ equals 0.7953.

## 4. Results and Discussions

In this section the analysis results of ionospheric correction based on NeQuick 2 adaption to the previous day GIMs will be displayed and discussed. The ability of NeQuick 2 to predict global daily TEC values will be assessed, and the results of an attempt to further improve the model performance will also be proposed.

Considering the situation that in some applications we cannot get GIMs or other measurements the day before, the possibility of using measurements taken at earlier times may have some practical meanings and is investigated here. In this context, we performed a test study of ionospheric correction given by NeQuick 2 adaption to GIMs data of the previous day, during 1 to 3 days before, during 1 to 5 days before, respectively. The results of zi_10_ can be considered as an indication of the model performance in predicting the TEC values at a given day after ingesting the GIMs data of a suitable number of days before. For comparison, the results of NeQuick 2 adaption to GIMs of the current day are also present.

**Figure 8:**
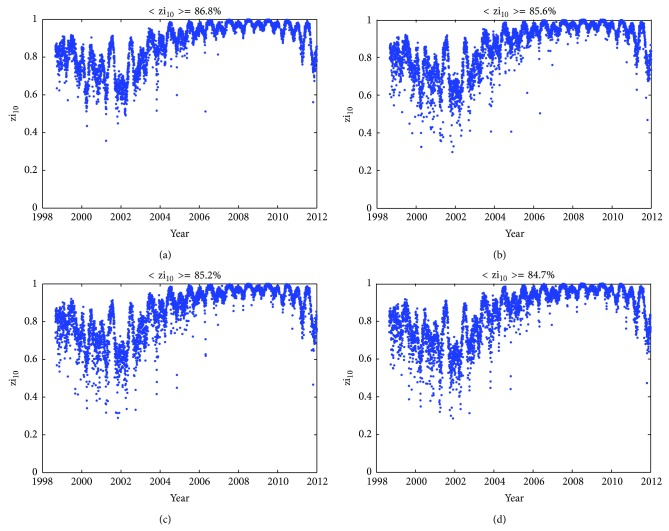
Time series of zi_10_ computed by ingesting of JPL GIMs into NeQuick 2 in a Galileo-like mode. The ingested GIMs data are of the current day (a), of the previous day (b), during 1 to 3 days before (c), and during 1 to 5 days before (d), respectively. The mean values of zi_10_ are given in the title.

**Figure 9:**
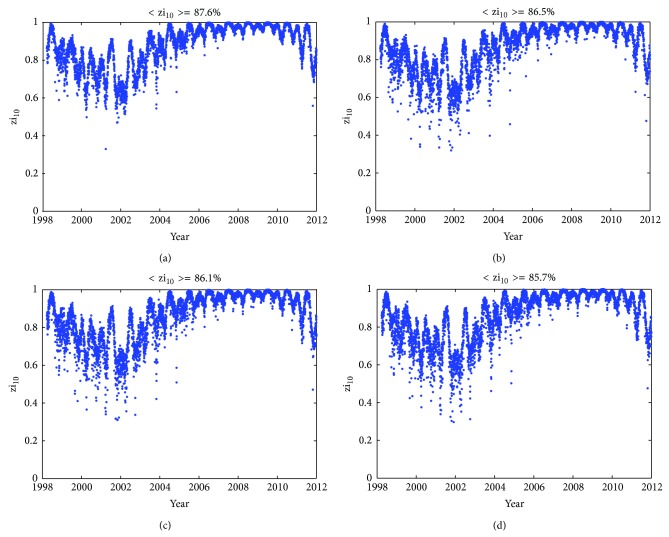
As Figure 8, but the ingested GIMs data are from CODE.

Time series of zi_10_ are given in Figure 8, computed by ingesting previous day(s) JPL GIMs into NeQuick 2 and Az are modeled with 3 coefficients according to (4), like in the scheme adopted by Galileo ICA. The ingested GIMs data are of the current day (a), of the previous day (b), during 1 to 3 days before (c), and during 1 to 5 days before (d), respectively. The mean values of zi_10 _are given in the title.  Figure 9 shows similar results but the ingested GIMs data are from CODE. From both figures, the following can be concluded. (1) In general, the performance of GIMs-driven-NeQuick model in predicting global TEC distribution is quite good. Few values of zi_10_ fall below 60%, and the mean values get close to 85%. (2) Very similar trends of zi_10_ changes with time can be seen quite clearly, regardless of GIMs from JPL or CODE, of GIMs from the current day or historical data as old as 5 days. The temporal variations of zi_10_ are over several time scales, such as annual, semiannual, and solar cycle periodic variations. (3) As more measurements from earlier days are used, the accuracy of NeQuick 2 adaption to both JPL GIMs and CODE GIMs may decrease. When NeQuick 2 is adapted to JPL GIMs, zi_10_ decreases from 86.78% using the current day GIMs to 85.61% using the previous day GIMs, then to 85.18% using GIMs 1 to 3 days before, and finally to 84.72% using GIMs 1 to 5 days before. When NeQuick 2 is adapted to CODE GIMs, zi_10_ generally shows similar trends but a little higher in terms of amplitude correspondingly. (4) The performance of GIMs-driven-NeQuick model also presents variability with solar activity and behaves better during low solar activity years. It can be seen from the zi_10_ values during the years 2007 to 2010 indicating that the TEC error is below 10 TECU 90% of the time.

As an attempt to further improve the accuracy of global daily TEC prediction, we add a 4th term to (4), to form the following expression:

(6) $$\begin{array}{c} \text{Az}=a_{0}+a_{1}\mu +a_{2}\mu^{2}+a_{3}\mu^{-2}. \end{array}$$

**Figure 10:**
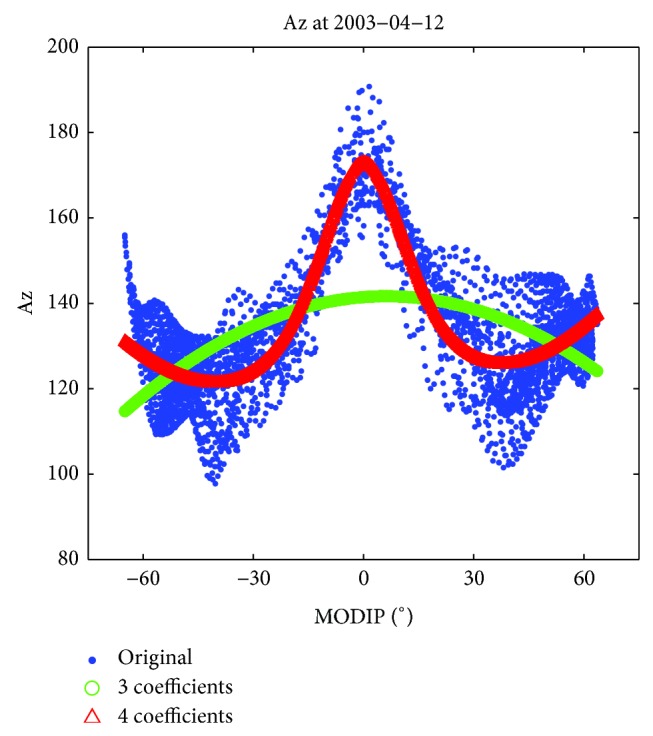
Az versus MODIP for 12 April 2003.

A justification for this representation is given after considering Figure 10, which presents an example of calculated Az values at every grid point (blue dots) versus MODIP and the corresponding Az fitting values obtained with (4) (green) and (6) (red). It is clear that the 4- coefficient function can reproduce the trends of Az values better than that of 3 coefficients. Particularly in the equatorial anomaly region, the peak is represented more precisely. According to Figure 10, the following is clear. (1) After adding the 4th coefficients, the wavelike shape of Az values can be reproduced well. (2) For a fixed MODIP, there is a spread of Az, and the variability of Az can reach up to 50 (near 40°N), and we attribute it to the longitudinal effect as shown in Figure 3. Thus we suppose that using a single value to characterize them will not be enough and induce some errors. (3) The global variability of Az can reach up to 100 and it clearly reflects that the NeQuick 2 model itself can not fully reproduce the detailed characteristics of global daily TEC distribution, in particular with the Galileo-like adaption technique.

When Az are modeled with 4 coefficients according to (6), time series of zi_10_ shows similar trends as that when Az are modeled with 3 coefficients according to (4), whether ingested previous day(s) GIMs data are from JPL or CODE. Due to the similarity, we do not display the individual time series of zi_10_ here, but only the mean values of zi_10_ and corresponding improvement are summarized in Tables 1 and 2.

From both tables, it is obvious that the performance of global daily TEC predictions is slightly improved further after adding the 4th coefficient. On average, this improvement is ~1.56% for JPL GIMs and ~1.37% for CODE GIMs, respectively.

It is interesting to note that even using GIMs dataset of the current day, the zi_10_ mean values can hardly reach to 90%. This is probably due to (1) the longitude variation of Az index; (2) the performance of NeQuick 2 model might have local time dependence at a selected location, thus only one Az index is not sufficient to reduce the residual error significantly; (3) the uncertainty of the observations used. The fact that zi_10_ may decrease as more measurements from earlier days are used in the constructing process may be due to the short correlation scale of ionospheric TEC. Thus, for forecasting purpose, historical data older than 5 days should not be considered. It has to be noted that the improvement of TEC predictions is not so remarkable as that of Az as shown in Figure 10. This fact may partly reflect that more efforts should be devoted to the model parametrization to improve the NeQuick capabilities in the equatorial and high-latitude regions, such as the electron density and TEC dependence on local time in these areas. The performance of ionospheric correction based on ingestion of GIMs dataset into NeQuick 2 also varies with seasons and geographic latitudes. A more detailed study on these aspects is being carried out and the relevant results will be present in the next future.

## 5. Conclusions

NeQuick is an empirical model that describes spatial and temporal variations of the ionospheric electron density. It employs the ionosonde observations derived ionospheric peak density and height maps as the basis and uses the DGR formulation to represent the altitude variation of electron density. The model output is the ionospheric electron density and TEC for the given location, time of the day, month, and solar activity. An adapted model version of the European Space Agency has been used in the Galileo single frequency receivers to compute ionospheric corrections. The effective ionization parameter input is described by three coefficients.

In this paper, we carried out a study of ionospheric modeling based on the NeQuick 2. Effective ionization indices (Az) have been estimated by ingesting observations into the model. In particular, GIMs computed by JPL and CODE during more than 13 years have been used. The investigation aims to assess the performance of NeQuick 2 in providing global TEC predictions after ingesting previous day GIMs data. The main conclusions are as follows.

1. In general, the performance of NeQuick 2 adapted to GIMs is quite good, with average 86% of vertical TEC error less than 10 TECU, when the global daily Az function of MODIP is constructed as a second order polynomial. The performance of NeQuick 2 adapted to GIMs presents variability with solar activity and behaves better during low solar activity years.
2. As more measurements from earlier days are used, the accuracy may decrease. Thus, for forecasting purpose, we propose historical data older than 5 days should not be considered. The accuracy of TEC predictions is improved further after adding a 4th coefficient to the original expression adopted by Galileo ionospheric correction algorithm, but not remarkably. More robust technique is needed in the future to accurately nowcast/forecast the ionosphere [37].

## Figures and Tables

**Table 1:** Mean values of zi_10_ and improvement, when JPL GIMs are ingested into NeQuick 2.

	With 3 coefficients	With 4 coefficients	Improvement
Current day	86.78%	88.45%	1.67%
1 day before	85.61%	87.15%	1.54%
1 to 3 days before	85.18%	86.70%	1.52%
1 to 5 days before	84.72%	86.22%	1.5%

**Table 2:** As Table 1, but the ingested GIMs are from CODE.

	With 3 coefficients	With 4 coefficients	Improvement
Current day	87.56%	89.03%	1.47%
1 day before	86.46%	87.80%	1.34%
1 to 3 days before	86.08%	87.42%	1.34%
1 to 5 days before	85.66%	86.98%	1.32%
